# Utilization of Biomass Waste from Citrus Fruits for the Production of Essential Oils

**DOI:** 10.3390/foods15081446

**Published:** 2026-04-21

**Authors:** Esmeralda Quilo Catucuamba, Jimmy Alba Lechón, Favian Bayas Morejón, Orlando Meneses Quelal, Juan Gaibor Chávez

**Affiliations:** 1 Carrera de Agroindustria, Facultad de Ciencias Agropecuarias, Recursos Naturales y del Ambiente, Universidad Estatal de Bolívar (UEB), Guaranda 020150, Ecuador; jquilo@mailes.ueb.edu.ec (E.Q.C.); jalba@mailes.ueb.edu.ec (J.A.L.); fbayas@ueb.edu.ec (F.B.M.); jgaibor@ueb.edu.ec (J.G.C.); 2 Carrera de Alimentos, Facultad de Industrias Agropecuarias y Ciencias Ambientales, Universidad Politécnica Estatal del Carchi (UPEC), Tulcán 40101, Ecuador

**Keywords:** essential oils, citrus peel, fractional distillation, kinetic modeling, Monod model, circular economy, limonene, biorefinery

## Abstract

The valorization of citrus peel residues represents an important strategy for promoting circular bioeconomy approaches in the agri-food sector. This study evaluated the biorefinery potential of ten citrus varieties cultivated in Bolívar Province, Ecuador, including mandarin (*Citrus reticulata criolla*, *Citrus nobilis* Loureiro, *Citrus tangerina*, *Citrus unshiu*), lemon (*Citrus aurantifolia Swingle*, *Citrus limonia*, *Citrus limonum*, *Citrus latifolia*), and grapefruit (*Citrus paradisi*, *Citrus paradisi* Macfad.), focusing on the extraction and characterization of essential oils from peel biomass. The residual biomass was characterized through proximate and elemental analyses to determine its physicochemical properties, and essential oils were extracted under two maceration times (8 and 12 h) to evaluate the influence of extraction conditions on yield. Chemical composition was determined by gas chromatography–mass spectrometry (GC-MS). The results revealed significant variability among varieties in moisture, ash, and volatile solids content. Citrus nobilis Loureiro showed the highest extraction yield, while grapefruit varieties exhibited the greatest increase in yield with extended maceration time. Limonene was identified as the predominant compound in all essential oils, reaching concentrations above 90% in grapefruit samples, and significant intervarietal differences in monoterpene profiles were observed. Extraction kinetics were evaluated using seven mathematical models, among which the Monod model showed the best fit to the experimental data (R^2^ > 0.99), demonstrating strong predictive capability. These findings highlight the potential of citrus peel residues as sustainable sources of high-value essential oils and provide a quantitative framework for optimizing extraction processes within citrus biorefinery systems.

## 1. Introduction

Citrus fruits, renowned for their wealth of bioactive and aromatic compounds, have been the subject of extensive scientific research thanks to their versatility and application in diverse industries, including food, pharmaceuticals, and cosmetics [1]. Within this vast landscape, citrus peel has emerged as an exceptionally rich source of essential oils with remarkable therapeutic and sensory properties [2]. In industrial settings, minimizing raw material losses and reducing waste generation are crucial imperatives for mitigating negative environmental impact [3]. Byproducts, including peels, seeds, and residual membranes of citrus fruits, represent approximately 50% to 60% of the total fruit weight in citrus processing operations [4]. In this context, the use of biomass waste from citrus fruits in obtaining essential oils has sparked growing interest in the scientific community due to its promise to mitigate the accumulation of organic waste and promote the sustainable production of highly valuable compounds [5].

The use of citrus fruit waste to obtain essential oils not only contributes to reducing organic waste but also offers the possibility of generating additional income for the agricultural industry and improving the environmental sustainability of citrus production operations [6]. This practice represents an innovative approach to addressing the global challenge of agricultural waste management while taking advantage of the valuable compounds present in citrus peel. As noted by Pomoni et al. [7], valorizing this waste stream not only mitigates the environmental impacts associated with its disposal but also generates new economic opportunities for agricultural producers. Furthermore, several studies have demonstrated the potential of essential oils obtained from citrus waste in a wide range of industrial applications, such as the food, pharmaceutical, and cosmetic industries [8]. In this context, ongoing research in this field is crucial not only to improve the extraction and valorization processes of these compounds but also for better understanding their environmental and economic impact throughout the entire citrus supply chain [9].

The production of essential oils from citrus peels benefits greatly from research on the bioactive compounds present in these peels [10]. Numerous studies have focused on exploring potential applications of citrus peel in the food industry, given its richness in various active components such as dietary fiber, pectin, proteins, pigments, flavonoids, and essential oils [11]. Studies conducted by Zárate et al. [12] have explored these compounds and developed effective extraction techniques to obtain them from citrus peel. Furthermore, research such as that of Liu et al. [13] and Panwar et al. [14] has demonstrated the wide range of applications of these bioactive compounds in various industries. These applications address a variety of biological properties, such as antioxidants, antimicrobial, anticancer, anti-inflammatory, and antidiabetic activities [15]. This knowledge supports and enhances the production of essential oils and antioxidants from citrus peels, providing a solid scientific basis for the selection of compounds and more effective extraction techniques [16].

The extraction of essential oils from citrus fruit biomass waste can be achieved through various techniques, such as hydrodistillation [17], solvent extraction, and cold pressing [18]. Hydrodistillation is an economical and efficient process for obtaining essential oils, in which saturated steam under pressure is used to extract volatile compounds from the plant material [19]. Solvent extraction involves the use of organic liquids to dissolve the desired compounds, while cold pressing uses mechanical pressure to extract the liquid from the plant material without the use of heat [20]. Among the various extraction alternatives, fractional distillation is emerging as a promising application, widely documented in the scientific literature [21]. This technique offers numerous advantages as it reduces the boiling point of the essential oil within the column. This temperature reduction mitigates the degradation of essential components, minimizing the adverse effects of high temperatures [22].

Despite the extensive research on citrus essential oil extraction, several limitations remain in the current literature. Most studies focus on single citrus species or evaluate a limited number of varieties under different experimental conditions, which restricts the possibility of performing comparative analyses of extraction efficiency and chemical composition. Furthermore, although kinetic modeling has been applied to describe mass transfer phenomena during the extraction of plant bioactive compounds, its systematic application to citrus essential oil extraction remains scarce, particularly in studies that compare multiple mathematical models under identical processing conditions. In addition, available information regarding the physicochemical properties and essential oil composition of citrus varieties cultivated in Ecuador is still very limited.

Therefore, the novelty of this study lies in the integrated evaluation of citrus peel biomass from ten citrus varieties cultivated in Ecuador, combining physicochemical characterization, essential oil extraction, GC-MS chemical profiling, and comparative kinetic modeling using seven mathematical models. By integrating compositional analysis with kinetic modeling, this study contributes to improving the understanding of citrus essential oil extraction dynamics and provides useful information for optimizing citrus biomass valorization strategies.

## 2. Materials and Methods

### 2.1. Plant Material

For this study, samples of four mandarin varieties were selected and collected: *Citrus reticulata* criolla (CRC), *Citrus nobilis* Loureiro (CNL), *Citrus tangerina* (CT) and *Citrus unshiu* (CU); as well as samples of four varieties of lemon: *citrus aurantifolia swingle* (CAS), *Citrus limonia* (CL_1_), *Citrus limonum* (CL_2_) and *Citrus latifolia* (CL_3_). In addition, samples of two grapefruit varieties were collected: *Citrus paradisi* (CP) and *Citrus paradisi* Macfad (CPM). These fruits were obtained from the cantons of the province of Bolívar, specifically from Echea (1°26′00″ S 79°16′00″ W), Caluma (1°38′00″ S 79°15′00″ W) and Las Naves (1°17′00″ S 79°18′00″ W).

### 2.2. Experimental Methodology

All experiments were performed using three biological replicates for each citrus variety. Each biological replicate consisted of an independent batch of citrus fruits collected from the study areas. For each biological replicate, technical replicates were conducted depending on the type of analysis. Proximate and elemental analyses were carried out in triplicate (n = 3). Essential oil extraction experiments were also performed in triplicate for each variety and maceration condition. Physicochemical measurements (density and pH) were conducted in triplicate. GC–MS analyses were performed using three independent injections for each essential oil sample to ensure chromatographic reproducibility. Reported values correspond to the mean ± standard deviation of the replicates.

#### 2.2.1. Sample Preparation

The citrus peels were cut into 1 cm^2^ pieces, weighing 300 g for mandarin varieties and 200 g for lemon and grapefruit varieties. These samples were placed in glass containers for maceration. A total of 250 mL of distilled water and 0.7% NaHCO_3_ (sodium bicarbonate, ≥99%, Sigma-Aldrich, St. Louis, MO, USA) were added per 100 g of sample. Once the plant material and reagents were placed in the containers, they were sealed and shaken for 1 min to homogenize the contents. The containers were then left to stand for 8 to 12 h. These specific durations were selected based on common practices reported in the literature for the pre-treatment of plant materials prior to essential oil extraction, aiming to promote sufficient hydration and partial cell wall disruption [23], while also exploring a relevant operational window for potential industrial applications. This pre-maceration procedure was carried out following previously reported methodologies designed to enhance the release of volatile compounds from citrus matrices prior to distillation [24]. All extraction experiments were performed in triplicate (n = 3) for each variety. The addition of NaHCO_3_ (0.7%) was intended to promote partial disruption of pectic substances in the flavedo, facilitating glandular oil release.

#### 2.2.2. Analysis of Citrus Peel

Proximate and elemental analysis of citrus fruit peels was performed to determine their chemical composition. The proximate analysis determined moisture content (UNE-EN ISO 18134-2:2017) [25], ash content (UNE-EN ISO 18122:2016) [26], and total volatiles (UNE-EN ISO 18123:2015) [27]. The elemental analysis measured the percentage of carbon, hydrogen, nitrogen, and sulfur according to UNE-EN ISO 15104 [28].

#### 2.2.3. Extraction of the Essential Oil

A fractional distillation apparatus (Biobase, Shandong, China) was used. This apparatus consists of a 2000 mL heating mantle that reaches a maximum temperature of 380 °C, a 2000 mL distillation flask, a 10-plate fractionating column, a distillation head or T-piece, an adapter with a mercury thermometer, a straight condenser, a distillation tail, an Erlenmeyer flask, and a separatory funnel. The extraction conditions were established based on previously reported distillation protocols for citrus essential oil recovery, with a focus on temperature to maximize monoterpene extraction while minimizing thermal degradation [29]. All tests were performed at a temperature of 78–85 °C, a mass flow rate of 9.03–16.81 mL/s, a pressure of 0.973 atm, and a time of 120 min. A 10 mL graduated cylinder (Pyrex, Corning, NY, USA) was used to measure the volume. The samples were stored in amber glass vials (Supelco, Bellefonte, PA, USA), which were then wrapped in aluminum foil to prevent exposure to light and kept at 12 °C until further chemical analysis.

##### Essential Oil Density

To determine the density of the essential oils of mandarin, lemon, and grapefruit, the samples were placed in a 10 mL pycnometer (Brand, Wertheim, Germany) at a temperature of 20 °C. The density was calculated using the following Equation (1):

(1) $$\rho =\frac{m_{2}-m_{1}}{v}$$

where

ρ: density of the essential oil, g/mL

*m*_1_: mass of the empty pycnometer, g

*m*_2_: mass of pycnometer + essential oil, g

v: volume of essential oil, mL

##### pH of Essential Oil

The pH measurement of the essential oil samples was performed using the pH meter (Hanna Instruments, Woonsocket, RI, USA).

##### GC-MS Analysis of the Essential Oil

The chemical composition of the essential oils of mandarin, lemon, and grapefruit was identified using a gas chromatograph (TRACE 1300) coupled to a single quadrupole mass spectrometer (ISQ 7000) (Thermo Fisher Scientific, Waltham, MA, USA) and a DB-5MS column (30 m long, 0.25 mm internal diameter, and 0.25 µm thick). The injector temperature was maintained at 230 °C using split injection mode with an injection volume of 1 µL. The oven temperature program was from 50 °C to 230 °C at a rate of 3 °C/min. The total running time was 66 min. Compound identification was performed by comparison of mass spectra with the NIST mass spectral library and by comparison of calculated retention indices with literature data [30].

For sample preparation, 3 µL of each essential oil was weighed into amber chromatography vials and 1 mL of nonane standard solution with cyclohexane was added.

Compound identification was carried out by comparing the obtained mass spectra with those available in reference spectral libraries (e.g., NIST/EPA/NIH Mass Spectral Library) and considering the corresponding retention times. When available, compound assignments were further supported by comparison with previously reported data in the literature. Each sample was analyzed in triplicate injections to ensure reproducibility of the chromatographic and mass spectrometric profiles.

The chemical diversity of the essential oil composition was evaluated using the Shannon diversity index ($H^{\prime }$). This index was calculated based on the relative abundance of each identified compound according to the following, Equation (2):

(2) $$H^{\prime }=-\sum p_{i}ln(p_{i})$$

where *p_i_* represents the proportion of each compound relative to the total chromatographic area of the essential oil. The index was used to compare the chemical diversity among the mandarin varieties analyzed.

The identification of volatile compounds was carried out by comparing the obtained mass spectra with those stored in the NIST mass spectral library and by comparing the calculated retention indices with values reported in the literature for similar chromatographic conditions. Retention indices were determined relative to a homologous series of n-alkanes analyzed under the same chromatographic conditions. Only compounds showing a spectral similarity higher than 80% and consistent retention index values were considered positively identified.

##### Experimental Yield of Essential Oil

In this study, different mathematical models were applied to determine the yield of essential oil extracted using fractional distillation. The yield of essential oils was estimated experimentally using the percentage yield equation [31] (Equation (3)).

(3) $$Y=\frac{v}{W}\times 100\%$$

where

*Y:* experimental yield of the essential oil (% v/*W*)

v: mass of essential oil (mL)

*W*: mass of the peel of the citrus fruits used (g)

Equation (3) allowed the calculation of performance values obtained from 10 min intervals.

##### Mathematical Modeling of the Yield and Extraction Rate of Essential Oils

In this study, different mathematical models were used, which were fitted to the experimental performance values. Seven models were used to determine which one provided the best fit. These mathematical models have been widely applied to describe biological growth and extraction kinetics in heterogeneous systems [32].

Monod mathematical model

(4) $$Y=\frac{Y_{max}\times t}{k+t}$$

Teissier’s mathematical model

(5) $$Y=Y_{max}\times \left(1-e^{\frac{-t}{k}}\right)$$

Haldane’s mathematical model

(6) $$Y=\frac{Y_{max}\times t}{k+t+t^{2}\times k_{i}}$$

Gompertz mathematical model

(7) $$Y=Y_{max}\times e^{\left({-e}^{-k*t+b}\right)}$$

Moser’s mathematical model

(8) $$Y=\frac{Y_{max}\times t^{n}}{k+t^{n}}$$

Powell’s mathematical model

(9) $$Y=\frac{Y_{max}\times t}{k+t+k_{i}}$$

Mathematical model of Logistic Law

(10) $$Y=Y_{max}\times \left(1-\frac{k}{t}\right)$$

where

*Y*: essential oil yield

$Y_{max}$: maximum yield, %

k: kinetic constant, min

$k_{i}$: constant, min^−1^

*t*: time, min

b: constant

n: constant

*t*: time, min

### 2.3. Statistical Analysis

All experimental measurements were performed in triplicate and results are expressed as mean ± standard deviation. Statistical analyses were conducted using R software (version 4.3.2; R Foundation for Statistical Computing, Vienna, Austria). Differences among citrus varieties and extraction conditions were evaluated using analysis of variance (ANOVA). When significant differences were detected, Tukey’s post hoc test was applied for multiple comparisons. Correlation analyses between variables were performed using Pearson’s correlation coefficient. Statistical significance was established at *p* < 0.05.

For the kinetic modeling analysis, model parameters were estimated using nonlinear least squares (NLS) regression implemented in RStudio (version 2023.12.1; Posit Software, Boston, MA, USA) using the nls () function in R (version 4.3.2; R Foundation for Statistical Computing, Vienna, Austria). Parameter estimation was performed by minimizing the residual sum of squares between experimental and predicted values through an iterative optimization procedure. Convergence was assumed when the relative tolerance was lower than 1 × 10^−6^, with a maximum of 100 iterations. Model performance was evaluated using the coefficient of determination (R^2^) and the root mean square error (RMSE). Confidence intervals (95%) for all kinetic parameters were estimated using the profile likelihood method implemented in the confint () function.

## 3. Results and Discussion

### 3.1. Chemical Composition of the Peel of the Citrus Fruits Mandarin, Lemon and Grapefruit

#### 3.1.1. Proximal Analysis

The results derived from the proximate and elemental analysis of mandarin, lemon, and grapefruit peels are summarized in Table 1. The proximate analysis of lemon, mandarin, and grapefruit peels provides a comprehensive evaluation of their chemical composition, including parameters such as moisture, ash, and total volatiles. A notable variability in moisture percentages (ω) emerged among the different citrus peel samples, with figures fluctuating approximately between 66% and 86%. This phenomenon suggests disparities in water-holding capacity among the various mandarin, lemon, and grapefruit varieties. In the lemon moisture results, variations were observed among the different varieties tested, with percentages ranging from 79.94% to 86.21%. Previous studies report moisture contents for citrus peels generally ranging between approximately 60% and 82%, depending on cultivar, environmental conditions, maturity stage, and postharvest handling practices. Reported values for mandarin and lemon peels commonly fall within the interval of 71–80%, confirming that the results obtained in the present study (74.90–79.48% for mandarin varieties and 79.94–86.21% for lemon varieties) are consistent with the variability documented for citrus biomass in previous investigations [33,34,35,36,37,38,39]. Such variability is frequently attributed to genetic differences among cultivars as well as agroclimatic conditions and physiological maturity at harvest. Regarding the results of the grapefruit peel moisture analysis in this study, the moisture content of *C. paradisi* was 69.787%, and that of *C. paradisi* Macfad was 66.049%. Moisture values reported for grapefruit peel in the literature generally range between approximately 70% and 81%, indicating a relatively high water content typical of citrus flavedo tissues. The values obtained in the present study (66.05–69.79%) are slightly lower than some previously reported ranges but remain within the expected variability for citrus residues, which can be influenced by cultivar characteristics, climatic conditions, and fruit maturity at harvest [40,41]. Regarding ash (As) percentages, these exhibited relatively modest levels in all samples, ranging between 4% and 5.5%.

The ash content of the different lemon varieties was: *C. aurantifolia swingle* 4.55%, *C. limonia* 5.48%, *C. limonum* 4.40% and *C. latifolia* 4.99%. Sadat et al. [42] and El-ghfar et al. [43] reported values of 6.26% and 6.58%, respectively, for the *C. aurantifolia* variety *Swingle*. Jiménez et al. [44] reported values of 3.69% in the *C. latifolia* variety. On the other hand, Akhtar et al. [45] reported an ash content of 3.39% for the *C. limonum* variety. In contrast, for mandarin citrus fruits, the ash content yielded results of 4.39% for the *C. reticulata* criolla variety and 5.37% for *C. nobilis*, 5.06% for *C. tangerina*, and 5.17% for *C. unshiu*. Studies conducted by Balderacchi et al. [46], Adeyanju et al. [47], and Xu et al. [48] obtained percentages of 0.48%, 3.96%, 2.88%, and 2.23%, respectively. In another study, Green et al. [49] reported percentages of 14.32%. Regarding grapefruit, the ash values were 4.436% for *Citrus paradisi* and 4.265% for *C. paradisi* Macfad. Kohajdová et al. [50], in a study on the characteristics of *C. paradisi* Macfad peel, reported values of 3.55%, while Edet et al. [51] reported values of 3.97%. On the other hand, Ahmad et al. [52] reported values of 6.24%.

The percentage of VS in the lemon varieties was as follows: *C. aurantifolia* Swingle, 84.45%; *C. slimonia*, 80.04%; *C. limonum*, 82.74% and 87.93%. In contrast, the VS percentages for mandarin were 82.33% for *C. reticulata* criolla and 88.03% for *C. nobilis* Loureiro, 90.25% for *C. tangerina*, and 88.22% for *C. unshiu*. Yankovsky et al. [53] reported lower VS values of 80.87% and 80.41% for the peel of Creole mandarin. Shin et al. [54] reported a VS value of 82.05% for tangerine peel. For unshiu mandarin peel, Wu et al. [55] reported a similar VS value of 88.14%. Finally, for grapefruit, VS values of 91.861% and 92.893% were obtained for *C. paradisi* Macfad. Cheong et al. [56] reported 97.96% VS for red grapefruit, while Yankovsky et al. [53], in their research on grapefruit peel, obtained 97.4% VS. The variation among results could be attributed to soil type, grapefruit variety, season, degree of maturity, and environmental conditions.

#### 3.1.2. Elementary Analysis

Regarding the elemental composition, the basic constituents of organic matter, a notable consistency was observed in the values of carbon (C), hydrogen (H), and nitrogen (N); however, sulfur (S) was not present in all analyzed samples. The elemental composition of citrus peel biomass reported in the literature generally shows relatively consistent proportions of carbon, hydrogen, and nitrogen, reflecting the lignocellulosic nature of these plant matrices. Previous studies indicate that carbon contents in citrus residues commonly range between approximately 39% and 56%, while hydrogen values are typically reported between 5% and 6.5%. Nitrogen contents are usually low, frequently below 1.5%, and sulfur is often absent or present only in trace concentrations. The elemental composition obtained in the present study (C: 39.03–42.07%; H: 6.09–6.52%; N: 0.71–0.95%; S: not detected) therefore falls within the variability reported for citrus peel biomass in previous investigations. Such variability is commonly attributed to differences in citrus species, environmental growing conditions, soil characteristics, and fruit maturity at harvest [57,58,59,60,61,62,63,64].

### 3.2. Extraction of the Essential Oil from the Citrus Fruits Used

The results of essential oil extraction using 8 and 12 h maceration (Table 2) show quantifiable patterns that integrate statistical aspects and scientific principles of secondary metabolite extraction. Statistical analysis of the data reveals an overall increase in extraction yield with increasing maceration time, with an average change of 0.207% and a mean percentage improvement of 14.77%. This trend aligns with the kinetic principles of extraction, where time is a determining factor in the diffusion of compounds from plant matrices [65].

A moderate negative correlation was observed between the initial yield Y (8 h) and the percentage change (r = −0.653), indicating that varieties with lower initial essential oil concentrations tended to show greater relative increases when the extraction time was extended. This phenomenon can be explained by kinetic models where the driving force (concentration gradient) is maintained for a longer time in matrices with lower initial concentrations, allowing for continuous extraction [65]. Conversely, in varieties such as CNL and CU with high initial yields, the system reaches equilibrium more quickly, resulting in smaller marginal improvements.

The high correlation between yields at 8 and 12 h (r = 0.973) indicates consistency in the efficiency ranking among varieties, with CNL maintaining the highest absolute yield (2.91%) followed by CU (2.61%). This consistency suggests that intrinsic factors of each variety, such as the density and distribution of essential oil glands in the flavedo, are primary determinants of extraction potential [66]. Histological studies have shown that varieties like CNL exhibit a higher density of schizogenous glands, which facilitates the release of volatile compounds during maceration [67].

The cases with the greatest improvement (CP: +45.2%, CPM: +40.3%) represent significant opportunities for industrial enhancement. These varieties show a particularly favorable response to the extended time, which could be attributed to specific membrane permeability characteristics or the presence of precursors that require prolonged times for complete hydrolysis and release [68]. In contrast, the observed decrease in CL_1_ (−16.4%) could be related to degradation or chemical recombination phenomena that warrant further investigation [69].

The remarkable stability of the physicochemical parameters (average Δρ = 0.002 g/mL, average ΔpH = 0.162 units) suggests that the basic chemical composition of the essential oils does not change significantly with extraction time. This stability is consistent with previous studies reporting high consistency in physical parameters during maceration extractions, indicating that the qualitative profile remains relatively constant [70].

From an industrial applications perspective, integrated statistical–scientific analysis provides a quantitative basis for strategic process decisions. Varieties classified as “high improvement” represent ideal candidates for processes where extraction time can be optimized to maximize yields, while varieties with high initial efficiency, such as CNL and CU, are preferable when cycle time is critical [71]. This time-response-based classification approach provides a systematic framework for varietal selection based on specific production objectives.

The synergy between statistical analysis and scientific rigor in this study contributes to the field of plant metabolite extraction by providing a quantitative framework for evaluating differential responses to extraction time. This integrated approach allows not only the description of trends but also the identification of underlying mechanisms and variety-specific improvement opportunities, advancing towards more efficient and sustainable extraction protocols [72].

### 3.3. Chemical Analysis of the Essential Oil of the Citrus Fruits Used

For consistency, the dominant chemical profile of each essential oil was used as the common descriptor across Table 3, Table 4 and Table 5. Detailed chemical analysis of citrus essential oils reveals complex compositional patterns that go beyond mere component identification, providing crucial quantitative information on intervarietal variability and its implications for functional properties. As presented in Table 3, statistical analysis of the data demonstrates that limonene is the main component in all varieties analyzed, with mean values of 84.99% ± 7.87% in mandarins (coefficient of variation, CV = 9.26%), 74.36% ± 9.53% in lemons (CV = 12.82%), and 91.44% ± 0.94% in grapefruits (CV = 1.03%). This statistically significant compositional variability (ANOVA, F = 4.85, *p* = 0.036) between taxonomic groups reflects profound biosynthetic differences that influence the organoleptic and bioactive properties of the oils [73].

Terpinene content in mandarins (r = −0.92, *p* < 0.05) suggests competitive biosynthetic regulation between these metabolic pathways, where high limonene synthase (LS) expression may suppress γ-terpinene synthase (GTS) activity. This inverse relationship is clearly manifested at the compositional extremes: CNL (92.98% limonene, 2.47% γ-terpinene) versus CRC (74.56% limonene, 20.21% γ-terpinene). Such compositional differences have direct implications for organoleptic properties, where γ-terpinene contributes secondary herbaceous and citrus notes that complement the primary character of limonene [74].

The quantitative analysis of the chemical components of the lemon varieties, presented in Table 4, reveals distinctive statistical patterns in the distribution of monoterpenes. The variability in the β/α-pinene ratio (0.00–5.18) among these varieties reflects differences in the specificity and relative activity of pinane synthases, particularly β-pinene synthase (β-PS), which shows differential expression among cultivars [75].

In the genus *Citrus*, the monoterpene biosynthetic pathway branches from the precursor geranyl pyrophosphate (GPP), where the enzymes pinane synthase (PS) and limonene synthase (LS) compete for the common substrate. The significant presence of β-pinene in CAS (22.71%) and CL_1_ (10.01%) confers enhanced antioxidant properties, as β-pinene exhibits greater free radical [76] scavenging activity than its α-isomer.

The chemical analysis of the grapefruits, detailed in Table 5, shows remarkable compositional homogeneity with a coefficient of variation of only 1.03%. This statistical consistency suggests stricter genetic regulation of the limonene pathway in these varieties, possibly associated with domestication and selection for consistent aromatic characteristics [77].

The high relative purity (>95%) in both cultivars indicates well-defined chemical profiles, particularly valuable for standardized applications in the fragrance and flavor industry, where compositional consistency is a critical quality parameter [78]. Multivariate correlation analysis between components reveals significant co-occurrence patterns that suggest coordinated regulation of related metabolic pathways. The positive correlation between sabinene and myrcene (r = 0.89) observed in several varieties could reflect the preferential activity of specific enzymes that catalyze sequential transformations in the acyclic [79,80] monoterpene pathway.

From a structure–activity relationship (SAR) perspective, statistically quantified compositional variability has direct implications for functional properties. Limonene, as the major component, contributes significantly to antioxidant activity through hydrogen donation and metal chelation mechanisms [81]. However, minor components such as γ-terpinene, linalool, and pinenes modulate these properties through synergistic effects, where the total antioxidant activity frequently exceeds the sum of the individual contributions [82].

The technological implications of these findings are multifaceted. For applications in the food industry, varieties with high limonene content and low chemical diversity (CNL, CP) are ideal for providing pure and stable citrus notes. In contrast, varieties with more balanced profiles (CRC, CAS) offer superior aromatic complexity for gourmet and high-end applications [83]. In the pharmaceutical and cosmetic sectors, the specific presence of linalool in CU (3.58%) and CT (2.36%) confers additional soothing and anti-inflammatory properties, expanding the spectrum of therapeutic applications [84].

The statistical–quantitative approach adopted in this analysis transcends traditional qualitative description, providing objective metric parameters for the characterization and classification of essential oils. The combination of measures of central tendency (means), dispersion (standard deviation, CV), correlation (Pearson coefficients), and diversity (Shannon index) constitutes a robust analytical framework for the comparative evaluation of chemical profiles in chemotaxonomic and quality control studies [85].

Statistically sound chemical analysis of citrus essential oils reveals a rich, quantifiable compositional diversity that reflects biosynthetic, genetic, and ecological differences among varieties. Integrating statistical parameters with biochemical and technological interpretations provides a solid foundation for the rational selection of plant materials for specific applications, thereby contributing to the development of higher value-added products in the food, cosmetic, and pharmaceutical industries.

### 3.4. Mathematical Modeling of the Yield and Extraction Rate of Essential Oils

**Figure 1:**
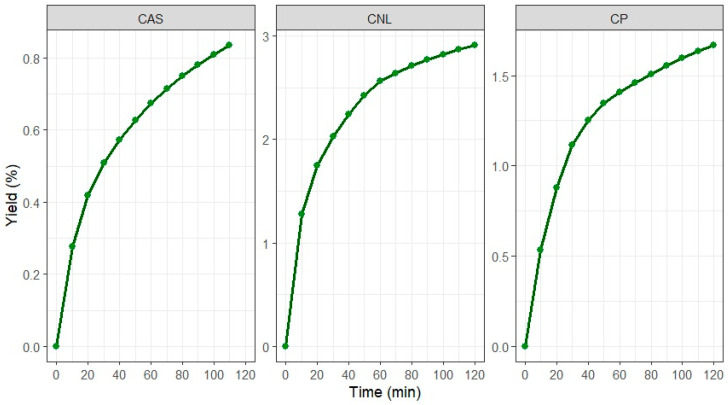
Essential oil yield of mandarin, lemon, and grapefruit varieties. Note: *C. nobilis* Loureiro (CNL), *C. aurantifolia swingle* (CAS), *C. paradisi* (CP), essential oil (EO). The weight of the peel used for the test was 300 g for CL, while for CAS and CP it was 200 g each.

The extraction of essential oils from plant matrices exhibits kinetics governed, at its different stages, by the interaction between structural characteristics of the substrate and the driving forces of transfer [86]. This is reflected in Figure 1, where the yield profiles as a function of time are non-linear and exhibit a clear transition: an initial phase of rapid release followed by progressive deceleration.

The essential oil yield curve is constructed from the mass of essential oil extracted relative to the amount of sample used. This curve provides a visual representation of how mass transfer occurs over time, which is crucial for understanding the efficiency of the extraction process. In this context, Figure 1 presents the yield at 10 min intervals for the best mandarin, lemon, and grapefruit treatments. A gradual increase in the amount of essential oil extracted is observed as the extraction time increases for all varieties studied. This increase can be attributed to the continuous diffusion of volatile components present in the citrus peel as the extraction process progresses [87]. Furthermore, variability in yield is observed among the different varieties, which may be related to differences in the chemical composition and physical properties of the citrus peels. This finding is consistent with previous studies that have shown that essential oil yield varies depending on the citrus variety and extraction conditions [88,89].

In the first few minutes (10–30 min), the steep slope indicates minimal surface resistance and almost immediate availability of volatile compounds in surface-distributed glands. This phenomenon is consistent with physical models of extraction from schizogenous glands, whose mechanical disruption (by maceration) maximizes the surface area to volume ratio and minimizes diffusion distances [90]. As time progresses, the slope decreases, reflecting a drop in the concentration gradient. This implies a shift to internal diffusive transport, where the microstructure (porosity, tortuosity, flavedo permeability) and the solubility of individual compounds become more relevant [91].

The fact that varieties like CNL (mandarin) exhibit higher initial yields and an early plateau suggests matrices with a higher accessible oil fraction and lower overall resistance, possibly due to thinner flavedo, higher gland density, or looser cell organization (“fragile matrices”) [92]. In contrast, grapefruit and lemon show a more prolonged growth rate and late plateaus, reflecting greater structural resistance and marked diffusive limitations.

Table 6 provides a detailed evaluation of several mathematical models applied to predict essential oil yield in the citrus extraction process. These models, including Monod, Teissier, Haldane, Gompertz, Moser, Powell, and the logistic law, were tested for experimental data from mandarin, lemon, and grapefruit varieties. These models were chosen due to their proven applicability in describing similar mass transfer phenomena in various biological and chemical systems, as well as their ability to capture different stages of the extraction process. Specifically:

The First Order and Second-Order models are fundamental kinetic descriptions useful for characterizing processes where the extraction rate depends on the concentration of extractable compounds or available sites.

The Elovich model is often applied to heterogeneous systems where energy activation changes during the process, suitable for complex plant matrices.

The Power Law model offers flexibility for complex kinetics, commonly observed in diffusion-controlled processes influenced by structural changes.

The Avrami model, originally for phase transitions, is relevant for describing release mechanisms from internal structures.

The Weibull model is a generalized empirical model known for its adaptability to sigmoidal and asymmetric release curves, common in biological materials with varied transport resistances.

The Peleg model is particularly apt for describing absorption/desorption kinetics in porous materials, directly relevant to solute diffusion in plant tissues.

By evaluating this comprehensive set of models, we aimed to identify the most suitable mathematical representation that captures the intricate mass transfer dynamics of essential oil extraction, providing insights into the rate-limiting steps and overall process behavior. Each model offers a unique perspective on how the extraction process behaves and how essential oil yield evolves over time.

The superiority of the Monod model is not accidental: this model describes systems in which the rate of a process (in this case, extraction) depends on a limiting substrate (accessible oil), capturing both the initial saturation and the progressive reduction in rate [93]. Its key parameter, k (time to half saturation), has a direct correlation with the overall resistance to mass transfer and the accessibility of active sites. The notable reduction in k in CNL supports the microstructural hypothesis discussed earlier.

The estimated kinetic parameters of the Monod model showed relatively narrow confidence intervals, indicating stable model fitting and good parameter identifiability. None of the 95% confidence intervals included zero, supporting the statistical significance of the estimated parameters.

The results show that each model has different capabilities in fitting the experimental data, as evidenced by the coefficients of determination (R^2^) and root mean square errors (RMSE). For example, the Monod model demonstrated a high coefficient of determination (R^2^) and a low RMSE for all three citrus varieties, suggesting that it fits the experimental data well and provides a good prediction of essential oil yield.

Based on the comparative statistical indicators presented in Table 6, the Monod model exhibited consistently high R^2^ values and low RMSE across the three citrus varieties. Therefore, this model was selected as the most appropriate to describe the extraction kinetics under the studied conditions

On the other hand, the Teissier model showed good predictive ability for mandarin and grapefruit, but lower accuracy for lemon, as demonstrated by the R^2^ and RMSE values. Similarly, other models such as Haldane, Gompertz, Moser, Powell, and logistic law offer varying degrees of fit to the experimental data, highlighting the importance of selecting the most appropriate model for each specific situation.

These results are consistent with previous studies that have used mathematical models to predict the yield of bioactive compounds in plant extraction processes. For example, Teleken et al. [94] used mathematical models to predict the yield of essential oils in the extraction of medicinal plants, finding that the choice of the appropriate model is critical to obtaining accurate predictions and enhancing extraction processes. o is critical to obtaining accurate predictions and improving extraction processes.

The parameter $Y_{max}$ represents the extraction limit under controlled experimental conditions. The close relationship between $Y_{max}$ modeling and experimentation suggests two things: first, that the systems operate close to thermodynamic equilibrium for the relevant process stages; second, that the observed kinetics are primarily determined by intrinsic properties of the raw material, and not by operational limitations associated with the extraction system. This point is crucial in food engineering and biorefinery operations, as it allows for greater confidence in scale-up, energy efficiency, and estimation of production capacities [95].

Beyond predictive capacity, the Monod model allows the process to be broken down into phases dominated by different mechanisms, facilitating the quantification of potential benefits by intervention in the microstructure or the use of assisted technologies (ultrasound, microwaves) that alter the resistance ratio, concepts easily addressed in heterogeneous systems from mathematical physics and computational simulation [96].

The fact that purely empirical (sigmoidal) models also achieve a good fit in some cases underscores a key principle: statistical fit does not equate to a mechanistic understanding of the process. Only those models whose parameters have physical meaning allow us to infer paths for process improvement and prediction outside the experimental range [97].

Understanding these behaviors and parameters makes it possible to make strategic decisions: selecting low k and high varieties $Y_{max}$; implementing pretreatments aimed at reducing internal resistance; or sizing stages that avoid unnecessary operating times, favoring the sustainability of the process [98].

The selection of the most appropriate model will depend on the nature of the extraction process and the specific characteristics of the experimental data. These models can be used by industry to optimize extraction processes and maximize the production of high-quality essential oils.

## 4. Conclusions

This study successfully demonstrated the effectiveness of non-thermal drying methods, specifically microwave vacuum drying (MVD), for optimizing the drying process of apple slices. MVD significantly reduced drying time and energy consumption compared to traditional methods, achieving efficient moisture removal while preserving key quality attributes. The kinetic analysis of apple slice drying revealed that the Peleg model provided the most accurate fit for the experimental data across all tested conditions, with high R^2^ values (above 0.99) and a low RMSE. This model effectively captured the moisture transfer phenomena during the drying process, indicating its suitability for predicting drying behavior under varying temperatures and microwave powers. The determined Peleg rate constant (k1) and equilibrium moisture content (k2) indicated a rapid initial drying phase and low final moisture content under optimal MVD conditions. These findings have significant implications for the food industry, particularly for fruit processing. Implementing optimized MVD protocols could reduce drying times by up to 50% and decrease energy consumption by 30% compared to conventional hot air drying, contributing to more sustainable and economically viable production of dried fruit products. Furthermore, the enhanced preservation of color, texture, and nutritional compounds observed with MVD offers a competitive advantage in the market for high-quality dried foods. Future research could focus on pilot-scale validation and a comprehensive techno-economic analysis of MVD for apple slices, as well as extending this approach to other fruit and vegetable matrices.

## Figures and Tables

**Table 1:** Proximate and elemental analysis of mandarin, lemon and grapefruit varieties.

Material Vegetable	Proximal			Elementary			
	ω (%)	As (%)	VS (%)	C (%)	H (%)	N (%)	S (%)
CRC	77.92	4.40	82.33	39.41	6.50	0.71	0
CNL	79.48	5.37	88.04	39.32	6.52	0.73	0
CT	74.90	5.06	90.25	39.03	6.09	0.75	0
CU	78.27	5.17	88.22	39.36	6.45	0.72	0
CAS	83.30	4.55	84.45	42.00	6.30	0.90	0
CL_1_	79.94	5.48	80.07	42.07	6.31	0.94	0
CL_2_	86.21	4.44	82.76	42.06	6.37	0.95	0
CL_3_	82.76	4.99	87.93	42.03	6.35	0.91	0
CP	69.79	4.44	91.86	41.38	6.36	0.71	0
CPM	66.05	4.27	92.89	41,43	6.38	0.71	0

Note: VS (volatile solids), C (carbon), H (hydrogen), N (nitrogen), S (sulfur), As (ash), yw (moisture), *C. reticulata* criolla (CRC), *C. nobilis* Loureiro (CNL), C. *tangerina* (CT) and *C. unshiu* (CU), *C. aurantifolia swingle* (CAS), *C. limonia* (CL_1_), *C. limonum* (CL_2_), *C. latifolia* (CL_3_), *C. paradisi* (CP) and *C. paradisi* Macfad (CPM).

**Table 2:** Extraction of essential oils from mandarin, lemon and grapefruit by maceration of 8 and 12 h.

Variety	Y (8 h) %	Y (12 h) %	ΔY (%)	Efficiency	*ρ* (8 h) g/mL	*ρ* (12 h) g/mL	Δ*ρ*	pH (8 h)	pH (12 h)	ΔpH
CRC	1.5	1.79	19:30	Moderate	0.84	0.84	0.00	5.12	4.87	−0.25
CNL	2.43	2.91	19.80	Moderate	0.83	0.84	0.01	4.63	5.48	0.85
CT	1.81	1.71	−5.50	Stable	0.84	0.83	−0.01	4.16	5.26	1.10
CU	2.53	2.61	3.20	Stable	0.83	0.83	0.00	4.98	4.94	−0.04
CAS	0.83	0.97	16.90	Moderate	0.85	0.85	0.00	4.42	4.43	0.01
CL_1_	0.67	0.56	−16.40	Low	0.85	0.85	0.00	4.43	4.43	0.00
CL_2_	0.69	0.81	17.40	Moderate	0.85	0.85	0.00	4.43	4.42	−0.01
CL_3_	0.72	0.84	16.70	Moderate	0.86	0.86	0.00	4.42	4.43	0.01
CP	1.15	1.67	45.20	High	0.85	0.84	−0.01	4.45	4.45	0.00
CPM	0.84	1.18	40.30	High	0.85	0.85	0.00	4.33	4.33	0.00

Note: ΔY = Percentage change in yield; Δ*ρ* = Change in density; ΔpH = Change in pH. Efficiency classification based on percentage change: High (>30%), Moderate (10–30%), Stable (−5% to 10%), Low (<−5%).

**Table 3:** Relative composition (%) of the main essential oil compounds identified in the analyzed samples by GC–MS.

Variety	Limonene (%)	α-Pinene (%)	γ-Terpinene (%)	Linalool (%)	Shannon Index *	Dominant Profile
CRC	74.56 ± 0.01	3.20 ± 0.03	20.21 ± 0.12	0.00	1.12	Limonene/γ-terpinene
CNL	92.98 ± 0.99	0.00	2.47 ± 0.06	0.00	0.25	dominant limonene
CT	86.48 ± 0.90	2.42 ± 0.05	5.82 ± 0.13	2.36 ± 0.06	0.51	Limonene with oxygenates
CU	85.94 ± 0.31	2.47 ± 0.00	5.08 ± 0.02	3.58 ± 0.01	0.58	Limonene-linalool

Note: * The Shannon index (*H*′) quantifies chemical diversity: low values indicate dominance of a few components, high values indicate greater compositional evenness. Only the major components are presented. The Shannon index was calculated only for mandarin varieties to evaluate the chemical diversity among these samples.

**Table 4:** Relative composition (%) of the main essential oil constituents identified by GC–MS in the evaluated samples.

Variety	Limonene (%)	β-Pinene (%)	α-Pinene (%)	γ-Terpinene (%)	β/α-Pinene Ratio	Dominant Profile
CAS	60.35 ± 0.33	22.71 ± 0.28	4.38 ± 0.06	9.14 ± 0.13	5.18	High in β-pinene
CL_1_	72.84 ± 0.13	10.01 ± 0.01	3.34 ± 0.00	10.76 ± 0.04	3.00	Pineal balance
CL_2_	82.50 ± 0.70	2.42 ± 0.03	2.57 ± 0.01	0.00	0.94	dominant limonene
CL_3_	81.75 ± 0.50	0.00	3.13 ± 0.02	7.58 ± 0.05	0.00	Limonene-γ-terpinene

Note: Only the major components are included in this table.

**Table 5:** Relative abundance (%) of the principal essential oil compounds detected by GC–MS.

Variety	Limonene (%)	α-Pinene (%)	Sabinene (%)	Myrcene (%)	Relative Purity * (%)	Dominant Profile
CP	92.10 ± 0.57	2.20 ± 0.00	2.70 ± 0.01	3.04 ± 0.00	96.8	Intense citrus, sweet
CPM	90.77 ± 0.09	2.32 ± 0.05	3.81 ± 0.07	3.10 ± 0.01	95.2	Fresh citrus, slightly bitter

Note: * Relative purity calculated as a percentage of major components (limonene + α-pinene + sabinene + myrcene). Minor compounds identified at trace levels are not included.

**Table 6:** Mathematical models tested to determine essential oil yield.

Model Name	CNL		CAS		CP	
	Parameter	Statistics	Parameter	Statistics	Parameter	Statistics
Monod	*k* = 17.8330 $Y_{max}$ = 3.3116	$r^{2}$ = 0.9950 RMSE = 0.0376	*k* = 32.0015 $Y_{max}$ = 1.0536	$r^{2}$ = 0.9934 RMSE = 0.0150	*k* = 25.3544 $Y_{max}$ = 2.0078	$r^{2}$ = 0.9973 RMSE = 0.0185
Teissier	*k* = 37.0709 $Y_{max}$ = 1.6308	r^2^ = 0.9256 RMSE = 0.1448	*k* = 41.4528 $Y_{max}$ = 0.4476	$r^{2}$ = 0.8103 RMSE = 0.0806	*k* = 37.2635 $Y_{max}$ = 0.9045	$r^{2}$ = 0.8341 RMSE = 0.1453
Haldane	*k* = 14.7416 $k_{i}$ = 0.0007 $Y_{max}$ = 3.0425	$r^{2}$ = 0.9975 RMSE = 0.0263	*k* = 20.6145 $k_{i}$ = 0.0018 $Y_{max}$ = 0.8322	$r^{2}$ = 0.9998 RMSE = 0.0029	*k* = 26.5949 $k_{i}$ = 0.0002 $Y_{max}$ = 2.0604	$r^{2}$ = 0.9832 RMSE = 0.0462
Gompertz	*k* = 0.0370 *b* = 0.1243 $Y_{max}$ = 2.9034	$r^{2}$ = 0.9959 RMSE = 0.0339	*k* = 0.0304 *b* = 0.3459 $Y_{max}$ = 0.8599	$r^{2}$ = 0.9922 RMSE = 0.0163	*k* = 0.0434 *b* = 0.4450 $Y_{max}$ = 1.6238	$r^{2}$ = 0.9857 RMSE = 0.0427
Moser	*k* = 12.1030 *n* = 0.7997 $Y_{max}$ = 3.6862	$r^{2}$ = 0.9990 RMSE = 0.0164	*k* = 21.6443 *n* = 0.7223 $Y_{max}$ = 1.4360	$r^{2}$ = 0.9995 RMSE = 0.0040	*k* = 0.9566 *n* = 0.0064 $Y_{max}$ = 0.0239	$r^{2}$ = 0.9002 RMSE = 0.1127
Powell	*k* = 8.9165 *n* = 8.9165 $Y_{max}$ = 3.3116	$r^{2}$ = 0.9950 RMSE = 0.0376	*k* = 16.0008 *n* = 16.0008 $Y_{max}$ = 1.0536	$r^{2}$ = 0.9934 RMSE = 0.0150	*k* = 12.6772 *n* = 12.6772 $Y_{max}$ = 2.0078	$r^{2}$ = 0.9973 RMSE = 0.0185
Logistic law	*k* = 6.3192 $Y_{max}$ = 2.8866	$r^{2}$ = 0.8998 RMSE = 0.1681	*k* = 7.5226 $Y_{max}$ = 0.7975	$r^{2}$ = 0.8505 RMSE = 0.0716	*k* = 7.5219 $Y_{max}$ = 1.6511	$r^{2}$ = 0.9241 RMSE = 0.0983

Note: *C. nobilis* Loureiro (CNL), *C. aurantifolia swingle* (CAS), *C. paradisi* (CP), coefficient of determination (R^2^), Root Mean Squared Error (RMSE), yield (Y).

## Data Availability

The original contributions presented in this study are included in the article. Further inquiries can be directed to the corresponding author.
